# Functional brain regeneration in the acoel worm *Symsagittifera roscoffensis*

**DOI:** 10.1242/bio.014266

**Published:** 2015-11-18

**Authors:** Simon G. Sprecher, F. Javier Bernardo-Garcia, Lena van Giesen, Volker Hartenstein, Heinrich Reichert, Ricardo Neves, Xavier Bailly, Pedro Martinez, Michael Brauchle

**Affiliations:** 1 Institute of Developmental and Cell Biology, Department of Biology, University of Fribourg, Chemin du Musée 10, Fribourg 1700, Switzerland; 2 Department of Molecular, Cell and Developmental Biology, University of California, Los Angeles, 621 Charles E. Young Drive, East Boyer Hall 559, Los Angeles, CA 90095-1606, USA; 3 Biozentrum, University of Basel, Klingelbergstrasse 50, Basel 4056, Switzerland; 4 UPMC-CNRS, FR2424, Station Biologique de Roscoff, Roscoff 29680, France; 5 Departament de Genètica, Universitat de Barcelona, A v. Diagonal, 643, Barcelona, Catalonia 08028, Spain; 6 Institució Catalana de Recerca i Estudis Avançats (ICREA), Passeig Lluís Companys, Barcelona, Catalonia 23 08010, Spain

**Keywords:** Brain regeneration, Xenacoelomorpha, *Symsagittifera roscoffensis*, Nervous system, Animal behavior

## Abstract

The ability of some animals to regrow their head and brain after decapitation provides a striking example of the regenerative capacity within the animal kingdom. The acoel worm *Symsagittifera roscoffensis* can regrow its head, brain and sensory head organs within only a few weeks after decapitation. How rapidly and to what degree it also reacquires its functionality to control behavior however remains unknown. We provide here a neuroanatomical map of the brain neuropils of the adult *S. roscoffensis* and show that after decapitation a normal neuroanatomical organization of the brain is restored in the majority of animals. By testing different behaviors we further show that functionality of both sensory perception and the underlying brain architecture are restored within weeks after decapitation. Interestingly not all behaviors are restored at the same speed and to the same extent. While we find that phototaxis recovered rapidly, geotaxis is not restored within 7 weeks. Our findings show that regeneration of the head, sensory organs and brain result in the restoration of directed navigation behavior, suggesting a tight coordination in the regeneration of certain sensory organs with that of their underlying neural circuits. Thus, at least in *S. roscoffensis*, the regenerative capacity of different sensory modalities follows distinct paths.

## Abstract (precis)

**Summary:** Brain and head regeneration in the acoel *Symsagittifera roscoffensis* is coordinated with restoration of directed navigation behavior, suggesting that the regenerative capacity of different sensory modalities follows distinct paths.

## INTRODUCTION

Regeneration of injured or lost tissue, organ or body parts is beneficial for animal survival. However, the capacity of their regeneration varies extensively between different animal species. While some invertebrate animal species, such as the planarian *Schmidtea mediterranea* or the cnidarian polyp *Hydra,* show astonishing whole-body regeneration from small tissue pieces, the regenerative capacity of other animal species, including humans, is rather limited (Li et al., 2015; Tanaka and Reddien, 2011). This difference in the regenerative capacity becomes particularly apparent in view of injuries of the central nervous system. For instance, surgical removal of brain tissue in humans (e.g. due to treatment of brain tumors) often severely impacts brain function and subsequent neurological recovery is rather due to a high degree of neuronal plasticity and rewiring than to the regeneration of brain tissue (Lepousez et al., 2015; Silver et al., 2015). In contrast, animals with high regenerative potential often show the striking ability to also regenerate their brain. In vertebrates, brain regeneration is well described in zebrafish, which shows the capacity to regenerate different regions of the brains, including the forebrain. While postnatal and adult neurogenesis has been described in many mammals only limited compensatory regeneration has been reported (Fernandez-Hernandez and Rhiner, 2015; Grandel and Brand, 2013). Notably, certain species of planarian and acoel worms are able to regenerate their head and brain after amputation. For example, it was recently reported that the acoel *Symsagittifera roscoffensis* is able to regrow its head and regenerate its brain within about 3-4 weeks after experimental decapitation (Bailly et al., 2014). Within the first few days after amputation a blastema forms at the site of injury. Subsequently head tissue starts to regrow and after a month the regenerated head is virtually indistinguishable from the one of intact animals. To what extent the regenerated brain acquires its original functionality however remains unknown.

While the organization of the nervous system of acoelomorphs appears to vary between different clades, it is typically composed of an orthogon with variable numbers of nerve cords and circular nerves, as well as an anterior brain defined by a condensed bilaterally symmetric neuropil associated with a high-density of neuronal cell bodies. Similar to plathyhelminth flatworms, the acoel orthogon is composed of a network of longitudinal bundles and interconnecting commissures (Achatz and Martinez, 2012; Bery et al., 2010; Raikova et al., 1998; Reuter et al., 1998, 2001; Semmler et al., 2010). The orthogon of *S. roscoffensis* contains three pairs of nerve cords that extend posteriorly from the brain and span the entire length of the body. By means of serial electron microscopy of an early juvenile specimen it was shown that *S. roscoffensis* at this stage comprises a centralized anterior brain made up of about 700 neurons (Bery et al., 2010). Associated with the brain are several sensory organs including a statocyst and a pair of eyes.

While the behavioral repertoire of most acoels remains poorly explored, *S. roscoffensis* has been reported to display strong and stereotypic responses to visual cues and gravity (Bailly et al., 2014; Keeble, 1910; Nissen et al., 2015). When exposed to a dark-light gradient or a directional light-scape *S. roscoffensis* shows a strong attraction to light. Similarly, when put on a tilted surface or in a water column *S. roscoffensis* crawls or swims downwards along the field of gravity. The sensory organ for light-perception is the eye, while gravity-sensing is presumably achieved by the statocyst.

Here we study the functional and neuroanatomical capacity of brain regeneration in *S. roscoffensis.* Since a detailed anatomical description of the adult *S. roscoffensis* brain is lacking we first established a neuroanatomical map of the neuropil domains of the mature brain. Our study indicates that the brain of the adult *S. roscoffensis* is comprised of defined commissural and longitudinal neuropil domains comparable to its organization as juvenile. The established neuropil map of the *S. roscoffensis* brain provides the possibility to assess the neuroanatomical organization of the brain after regeneration and further provides a reference for future studies that involve mapping distinct neuron types for instance marked by neurotransmitter expression.

To probe the degree of functional nervous system regeneration, we assessed different behaviors after decapitation and at different time points during head regeneration. We first demonstrated that the animal displays a robust and stereotypic body contraction behavior to mechanical vibration stimulation. Interestingly this behavior remained unchanged after decapitation, showing that it does not require a brain but is likely mediated by local sensory perception and processing within the orthogon. We further assessed phototaxis and geotaxis. Interestingly, phototaxis was restored within 3-5 weeks after decapitation suggesting that the eyes and visual system brain circuits fully regenerate (Yamasu, 1991). However, no recovery of geotactic behavior was observed within 7 weeks after decapitation, even though the statocyst itself had regenerated. This finding suggests that while superficially this sensory organ and the brain recover, the underlying neural circuits may either not be functionally reestablished, or alternatively, the regeneration of these may require a longer period to fully recover. Taken together, the regenerative capacity of the *S. roscoffensis* head, sensory organs and brain shows that complex behaviors can be recovered within weeks after decapitation providing an interesting system to study the recovery of functional brain circuits.

## RESULTS

### Anatomical organization of the nervous system and brain

**Fig. 1.**
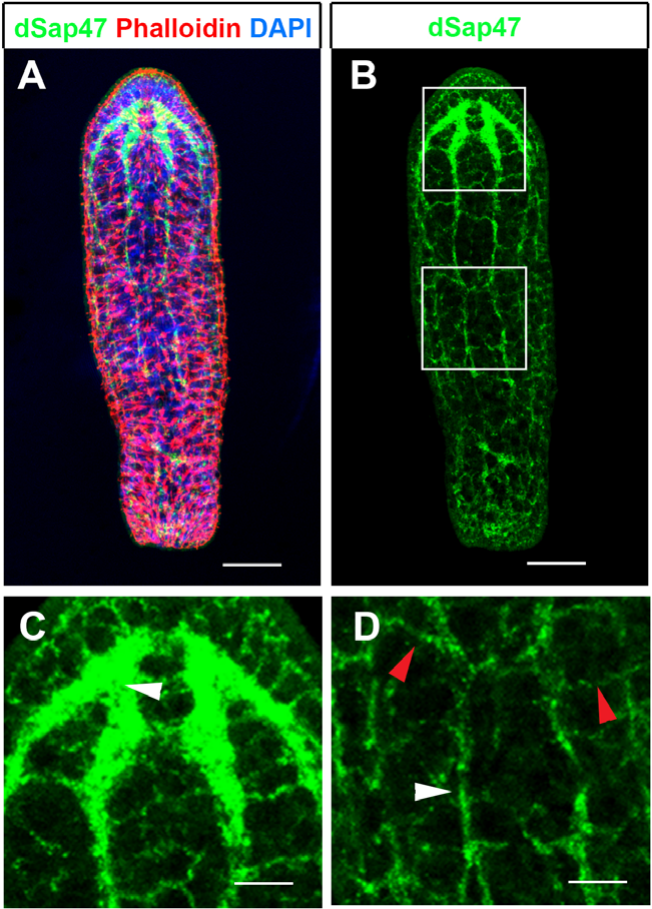
**Overview of the nervous system of the adult *S. roscoffensis***. (A,B) Maximum projection images of confocal microscopy stack of a specimen stained with anti-dSap47 (green), Phalloidin (red) and DAPI (blue). Insets shown in white boxes in (B) are shown in (C) for upper box depicting the brain, and (D) for lower box depicting a part of the peripheral nervous system. (C) Dense neuropil of the brain visualized by anti-dSap47 (green), (D) branches of nerve fibers of longitudinal cords (white arrowhead) and interconnecting commissures (red arrowhead). Scale bars: 80 µm in A,B; 40 µm in C,D.

In order to study brain regeneration in *S. roscoffensis* we first set out to establish an anatomical map of the neuropil domains of the adult. To establish anatomical markers for the neuropil we tested commercially available antibodies against synaptic proteins. We focused on antibodies of synaptic proteins since these highlight synapse dense regions of the brain and thus have been shown in many instances to provide essential landmarks to recognize and define the neuropil in more detail (Bressan et al., 2014; Dreyer et al., 2010; El Jundi et al., 2009; Heinze and Reppert, 2012; Perea-Atienza et al., 2015). We found that the antibody raised against the *Drosophila* synaptic protein 47 (dSap47) showed a strong and specific staining in the neuropil of brain, nerve cords and peripheral nerve net of adult *S. roscoffensis* (Fig. 1A-D) (Reichmuth et al., 1995)*.* The nervous system of *S. roscoffensis* has been shown to consist of an anterior brain, which is connected to six posteriorly running nerve cords. Nerve cords are interconnected by commissures as transverse fiber bundles forming a nerve net, termed orthogon (Bery et al., 2010; Perea-Atienza et al., 2015; Semmler et al., 2010). In agreement with this we find that anti-dSap47 immunostaining highlights the central neuropil of the brain (arrowhead in Fig. 1C) as well as the nerve cords (white arrowhead in Fig. 1D) and commissures between the nerve cords (red arrowhead in Fig. 1D).

**Fig. 2.**
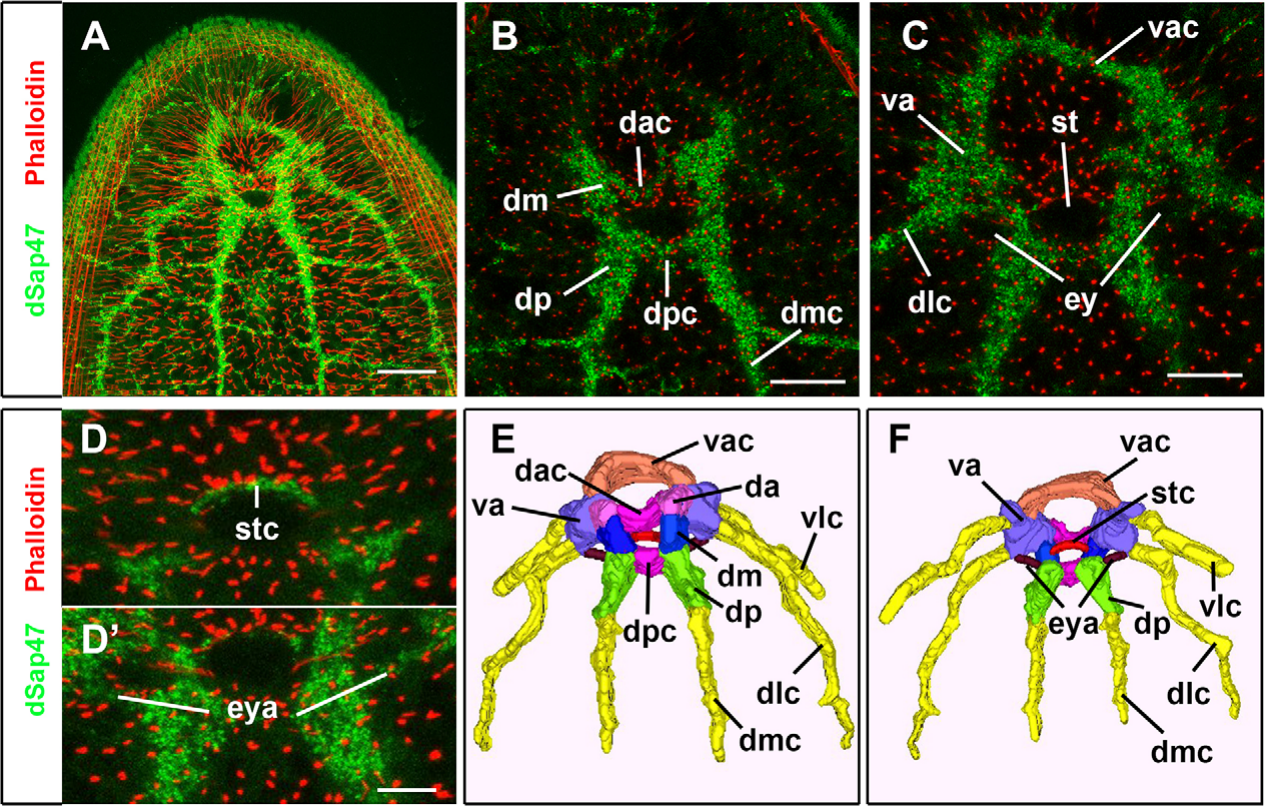
**Anatomical description of the neuropil domains of the adult *S. roscoffensis* brain**. (A) High-resolution image of the *z*-projection of a confocal stack of the entire brain and single dorsally (B) and ventrally (C) located sections showing the respective neuropil domains. (D-D′) Sensory organ associated neuropils are shown in D for the statocyst associated commissural neuropil and D′ for the eye-associated neuropil. (E,F) A 3D model representing the different neuropil domains in different colors is shown in E in a dorsal view and in F in a ventral view. Abbreviations: dorsomedial cord (dmc), dorsolateral cord (dlc), ventrolateral cord (vlc), dorsal posterior commissural neuropil (dpc), dorsal anterior commissural neuropil (dac), ventral anterior commissural neuropil (vac); statocyst associated commissural neuropil (stc), dorsal anterior neuropil (da), dorsal medial neuropil (dm), dorsal posterior neuropil (dp), ventral anterior neuropil (va), eye-associated neuropil (eya). Color coding: all nerve cords are shown in yellow, dpc and dac in pink, vac in orange, stc in red, da in purple, dm in blue, dp in green, va in dark purple and eya in dark red. Scale bars: 40 µm in A; 20 µm in B,C; 12 µm in D,D′.

We next characterized and annotated the brain neuropil using major anatomical landmarks to determine distinct domains (Fig. 2A-F). These major anatomical landmarks are the location where commissures emerge from the laterally running neuropil, the sites of emergence of nerve cords as well as the position of eye and statocyst. The neuropil organization is documented in single confocal sections of representative neuropil positions (Fig. 2B-D) shown in conjunction with a 3D model of the entire central nervous system (CNS) containing neuropil domains in different colors (Fig. 2E,F).

Three bilateral symmetric nerve cords extend posteriorly from the brain. These cords have previously been described as dorsomedial cord (dmc), dorsolateral cord (dlc) and ventrolateral cord (vlc) (Bery et al., 2010) (Fig. 2B,C,E,F). The boundaries between dlc or vlc and brain were defined as the point where they merge into large neuropil domains. Neuropil domains of the brain were annotated according to their relative location along the anterior-posterior and dorsal-ventral body axis. Three major neuropil domains span the midline forming commissures connecting the right and left hemispheres of the brain. These domains are the dorsal posterior commissural neuropil (dpc), the dorsal anterior commissural neuropil (dac) and the ventral anterior commissural neuropil (vac) (Fig. 2B,C,E,F). A fourth smaller commissural neuropil is located directly anterior to the statocyst, which we named statocyst associated commissural neuropil (stc) (Fig. 2D,F).

Furthermore we define three dorsal neuropil domains along the longitudinal axis: the dorsal anterior neuropil (da), dorsal medial neuropil (dm) and dorsal posterior neuropil (dp) (Fig. 2B,E). The posterior boundary of the dp neuropil is delimited by the anteriormost commissure between dmc and dlc (Fig. 2B). Medially to the bilaterally symmetrical dp compartments is the dpc, which also defines the boundary between the dp and dm neuropil (Fig. 2B,E). The anterior boundary of the dm neuropil is defined by the contact point between the dac and the da neuropils (Fig. 2B,E). The ventral anterior (va) neuropil is located ventrally to the da neuropil (Fig. 2C,F). The border between da and va neuropils is medially defined by the entry point of the dac and laterally by the emergence of the dlc (Fig. 2E,F). The anterior border between va and vac is defined by the emergence of the vlc (Fig. 2F). In addition, two eye-associated (eya) neuropils are found posteriorly to the eye connecting the dp with the va neuropil (Fig. 2D′,F). Taken together, the dSap47 staining robustly delimits the *S. roscoffensis* brain neuropil and allows us to demarcate major regions in order to reconstruct a brain map that can be used in future studies.

### Anatomical organization of the regenerated brain following decapitation

**Fig. 3.**
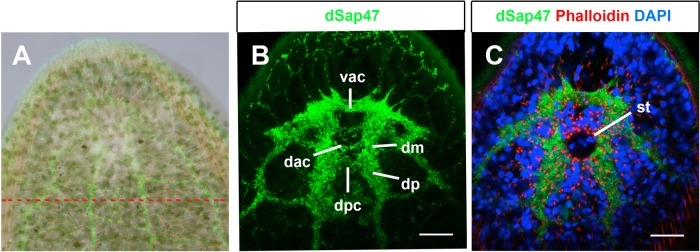
**Neuroanatomical description of brain regeneration.** (A) Approximated overlay of head photograph and brain confocal image depicting the line of decapitation experiments. The entire brain, parts of the nerve cords and all head sensory organs are thereby removed. (B,C) Representative specimen of complete regeneration of 70% of animals after 50 dpa of the neuroanatomical characterization of anti-dSap47 immunostaining (green), counter stained with Phalloidin (red) and DAPI (blue). Brain regeneration is virtually indistinguishable of non-treated animals. Abbreviations: dorsal posterior commissural neuropil (dpc), dorsal anterior commissural neuropil (dac), ventral anterior commissural neuropil (vac), dorsal medial neuropil (dm), dorsal posterior neuropil (dp), statocyst (st). Scale bars: 45 µm in B,C.

The striking ability of *S. roscoffensis* to regenerate its head after decapitation raises the question to what degree the anatomical organization of the brain is restored during the regeneration process. To investigate this, we decapitated animals and allowed them to regenerate for a period of up to 50 days. Decapitation was performed manually using a pair of sharpened minutien pins cutting the head posterior to the statocyst and anterior to the mouth opening. In this manner the entire head containing the brain, both eyes and the statocyst was surgically removed (Fig. 3A). Decapitation is an impacting procedure and a fraction of animals do not initiate head regeneration; these animals were isolated and excluded from further investigations. Of 50 decapitated animals 44 survived the first day; 42 initiated head regeneration. Most animals started to regenerate their head already at 5 days post amputation (dpa) in agreement with previously published observations (Bailly et al., 2014).

To document the full extent of regeneration of the brain in neuroanatomical terms, we sacrificed animals after 50 dpa and stained them with anti-dSap47 antibody to visualize the brain neuropil. We found that in about 70% of the animals brain regeneration occurred to a degree that the overall organization of the central brain neuropil domains was virtually indistinguishable from animals without brain amputation (Fig. 3B,C). The remaining 30% showed deformations particularly in the regeneration of commissural neuropil domains and the anterior brain (data not shown). Interestingly, independent of the degree of brain regeneration in all examined specimen the statocyst was regenerated (Fig. 3C).

### Decapitation does not alter the vibration response

**Fig. 4.**
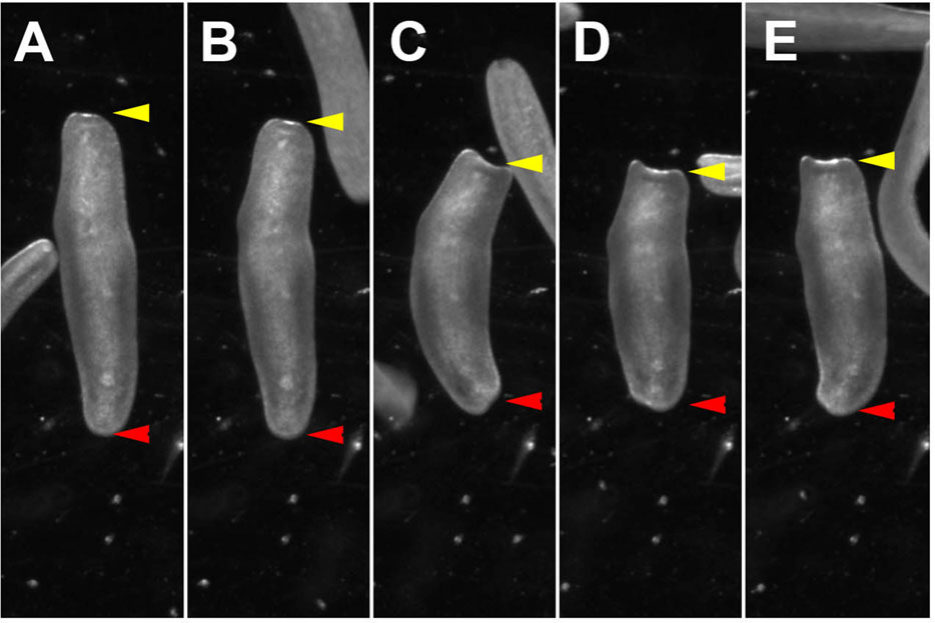
**Representation of vibration induced body contraction behavior.** (A-E) Temporal series of images showing the changes in body shape during the vibration induced body contraction behavior, (B) starting by stop of locomotion and stalling, (C-E) increasing contraction along the anterior-posterior body axis. Time between images about 0.2 s. Red arrowheads indicate the tail, yellow arrowheads indicate the head.

To test the functional capacity of brain regeneration we decided to study several specific behaviors in head-regenerated animals. The first of these was a vibration response to mechanical stimuli. For this we used a simple vibration assay, in which the animals were placed in a Petri dish under a stereoscope to visually score the behavioral response. A weak vibration, induced by tapping the walls of the Petri dish using a pipette tip, elicits a robust startle response in control animals, i.e. specimens which were not decapitated. The response includes a sequence of behaviors during which animals first stop moving and then contract their body along the anterior-posterior axis (see Materials and Methods). An image sequence of this behavior is shown in Fig. 4A-E (the red arrowhead points to the tail, the yellow arrowhead points to the head). We scored the presence or absence of the vibration response. The behavior is highly robust and stereotypic; 100% of the animals tested showed a vibration response. Typically animals initiate forward locomotion within seconds after the body contraction. A repeated vibration only seconds later is sufficient to elicit the next vibration induced body contraction. We did not further assess if habituation or sensory adaptation occurs, however, if it is the case it appears only to have a minor effect on the performance of the animal.

We tested the vibration response 5 dpa. Strikingly, we found that again 100% of the animals that recovered showed the same vibration response as before decapitation (Table S1). It appears unlikely that in 5 days a primordial brain has regenerated, suggesting that vibration induced body contraction is a somatosensory reflex processed by the orthogon. To test this, we repeated the experiment and tested the vibration response immediately after head amputation. Again we observed the same vibration response even immediately after amputation (Table S1). Thus indeed the animal does not require a head or brain to display a body contraction behavior in response to external mechanical stimuli. These results suggest that the sensory stimulus leading to the vibration response is perceived by mechanosensory neurons along the body. To further test if even shorter parts of the posterior body can display the vibration induced body contraction we performed a second experiment, in which we cut animals in the middle. Similar to the previous experiments we found that the posterior half, only containing parts of the orthogon and severed nerve cords, showed 100% response to vibration (Table S1). Moreover, also the anterior half, containing the head and brain with parts of the orthogon, had the same response (Table S1). This experiment shows that even shorter sections of the body, where only local circuitry of the orthogon remains, are sufficient to mediate a vibration induced body contraction.

Taken together, our data suggest that vibration induced body contraction does not require a functional brain and likely requires only local circuitry. In contrast, more complex behaviors such as directed navigation are likely to depend on sensory structures in the head as well as on sensory processing occurring in the brain.

### Phototaxis but not geotaxis is restored during head regeneration

**Fig. 5.**
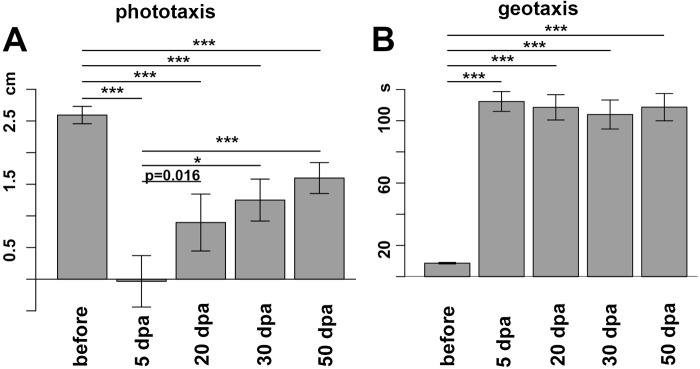
**Phototaxis and geotaxis behavior before and after decapitation.** (A) Phototaxis behavior before decapitation reaches a light-preference of 2.59 cm (s.e.m.=0.06 cm), at 5 dpa phototaxis was lost (0.0 cm light-preference, s.e.m.=0.26 cm). At 20 dpa, 30 dpa and 50 dpa a restoration of phototaxis occurs with a light-preference of 0.9 cm (s.e.m.=0.31 cm; not significant: *P*=0.016); 1.3 cm for 30 dpa (s.e.m.=0.34 cm; *P=0.002) and 1.6 cm for 50 dpa (s.e.m.=0.20 cm; ***P=3.57×10^−5^) when compared to 5 dpa. (B) Geotaxis behavior before decapitation reaches an average time of 8.6 s (s.e.m.=0.23 s) to move 1.7 cm at 45°. After amputation no geotaxis was observed (average time 112.3 s; s.e.m.=3.8 s; *P*=2.20E-16. At 20 dpa, 30 dpa and 50 dpa no recovery of geotaxis was observed (109 s at 20 dpa; s.e.m.=5.03 s ***P=2.20×10^−16^; 104 s at 30 dpa, s.e.m.=7.85 s, ***P=1.5×10^−11^; 109 s 50 dpa, s.e.m.=7.78 s ***P=5.19×10^−11^).

While the sensory repertoire of acoels remains largely unexplored, two well-known head sensory system based behaviors in *S. roscoffensis* are phototaxis and geotaxis (Keeble, 1910). Here we developed a light-gradient assay to measure phototaxis. Animals were put in the middle of a Petri dish with 6 cm in diameter illuminated by a white light gradient ranging from 1100 lux to 400 lux. A group of about 20-30 animals was placed in the middle of the gradient at about 840 lux and they were allowed to freely move around for 5 min. At this point the position of the worms within the light gradient was recorded. Before decapitation we measured positive phototaxis with an average of 2.59 cm (s.e.m.=0.06 cm, *N*=67) (Fig. 5A). To ensure recovery after decapitation we started to investigate this behavior 5 days after decapitation (5 dpa) but we did not observe phototaxis: the average positive phototactic displacement in the light gradient was 0.0 cm (s.e.m.=0.26 cm, *N*=37) suggesting that the animals moved randomly in the Petri dish (Fig. 5A). To cover the time frame during which brain regeneration occurs, we tested the same group of animals at 20 dpa, 30 dpa and 50 dpa. At 20 dpa a not significant increase of phototaxis was observed when compared to 5 dpa with an average positive phototactic displacement of 0.9 cm (s.e.m.=0.31 cm; *P*=0.016, *N*=31). Average positive phototactic displacement increased significantly at 30 dpa with 1.3 cm (s.e.m.=0.34 cm; *P=0.002, *N*=18) and 50 dpa with 1.6 cm (s.e.m.=0.20 cm; ***P=3.57×10^−5^, *N*=23) when compared to 5 dpa. However, during the 50 days of head and brain regeneration the original performance before amputation was not reached. There is also no significant increase of phototaxis between 20 dpa and 50 dpa (*P*=0.25).

To assess the recovery of general locomotion after decapitation we carefully observed recovering animals. Within the first hours after head amputation most animals show slow forward movement, generally displaying large circular motions, indicating that forward movement can occur in the absence of a brain. To quantify the level of locomotor recovery after decapitation we recorded the speed of control animals and compared it to the speed of animals 1 day after decapitation (see material and methods). The average speed of control animals was 1.63 mm/s (s.e.m.=0.048; *N*=77), while the average speed of animals at 1 day after decapitation was 1.37 mm/s (s.e.m.=0.061; *N*=89) significantly lower than before amputation (Fig. S1). In both groups however, there were also animals moving at more than 2.2 mm/s suggesting that there is no general locomotion defect (data not shown). Thus, while the average speed one day after decapitation is reduced to 84% as compared to before decapitation the defect in navigation cannot be attributed to an insufficient locomotion behavior.

Since *S. roscoffensis* lives on sand within the upper tidal zone the ability to move into the substrate and hide may be an important feature for survival. Indeed in nature animals are often found in aggregates on the surface of wet sand and mechanical disturbance (e.g. pressure by hand to sand surrounding an aggregate of animals) leads to a rapid disappearance of animals from the surrounding sand surface (Keeble, 1910). Also in the laboratory when put on a tilted surface the animal crawls downwards towards the bottom of a vessel. We therefore tested geotactic behavior of *S. roscoffensis* before and after decapitation. We used a simple geotaxis assay in which the animal was sucked into a pipette tip that was kept at approximately a 45° angle; after the animal settled in the pipette tip we marked the time that the animals needed to reach the bottom from a 1.7 cm mark (A maximum observation time of 2 min (120 s) was set in case the animal did not geotax). We first tested animals before head amputation and found that they required on average 8.6 s (s.e.m.=0.23 s, *N*=69) to reach the bottom (Fig. 5B). At 5 dpa we did not observe geotaxis with an average time of 112.3 s, since the observation time was stopped at 120 s only 2-3 animals reached the bottom (s.e.m.=3.8 s; *P*=2.20×10^−16^, *N*=42). To further test if geotaxis can be restored we tested animals at 20 dpa, 30 dpa and 50 dpa. The time to perform the task was 109 s at 20 dpa (s.e.m.=5.03 s, *N*=39), 104 s at 30 dpa (s.e.m.=7.85 s, *N*=21) and 109 s 50 dpa (s.e.m.=7.78 s, *N*=19), all significantly different from before head amputation (***P=2.20×10^−16^; ***P=1.5×10^−11^; ***P=5.19×10^−11^) and not significantly different from each other (5 dpa vs 20 dpa: *P*=0.60; 5 dpa vs 30 dpa: *P*=0.28; 5 dpa vs 50 dpa: *P*=0.82; 20 dpa vs 30 dpa: *P*=0.57; 20 dpa vs 50 dpa: *P*=0.85; 30 dpa vs 50 dpa: *P*=0.53). These experiments show that while phototactic behavior has partially recovered at 20 days after decapitation geotactic behavior does not become restored within 50 days after decapitation. Since animals were sacrificed at 50 dpa, geotaxis beyond this time point was not recorded. Interestingly, while geotaxis could not be behaviorally restored we found that even in extreme cases of abnormal head regeneration the statocyst was regenerated (data not shown).

## DISCUSSION

### Behavioral repertoire of *S. roscoffensis*

The behavioral repertoire of Xenacoelomorpha remains largely unexplored. Due to its abundance and symbiotic relation with the green algae *Tetraselmis convolutae,* the acoel *S. roscoffensis* had become a center of attention for studies from various fields already over a century ago (Bailly et al., 2014; Nissen et al., 2015). Frederick Keeble dedicated the first half of his book ‘Plant Animals - *a study in symbiosis*’ to the behavior of *S. roscoffensis* (at this time *Convoluta roscoffensis*). Already then it was noted that phototaxis and geotaxis are key behaviors in navigation. The two sensory organs providing input for these behaviors, eyes and statocyst, are characteristic features of the *S. roscoffensis* head. If eye and statocyst provide additional sensory information required for additional types of behavior other than directed navigation remains unknown. While it is reasonable to assume that phototactic behavior allows the animal to find light-exposed areas to improve the photosynthetic activity of its symbiont, it was recently shown that red light, which would be optimal for the photosynthetic activity of the algae, is not attractive to the animal (Nissen et al., 2015).

Sensory receptor neurons are found distributed everywhere along the epidermis of *S*. *roscoffensis*, they appear highly concentrated around the opening of the frontal gland (Bery et al., 2010). What cues these sensory neurons perceive remain unknown, but it appears likely that they also include chemosensory neurons since *S*. *roscoffensis* are also able to navigate in response to chemosensory cues (S.G.S and M.B., unpublished). It will be interesting to further probe for the spectrum of chemosensory behaviors, to identify attractive and repulsive cues and to test if these behaviors are dependent on the head and if they can be recovered after decapitation. Sensory neurons in acoels have been distinguished by ultrastructural features, such as the rootlet apparatus and specializations of the apical membrane (Bery et al., 2010; Todt and Tyler, 2007). However, how many molecularly distinct sensory neurons exist and what sensory cues they perceive remains unexplored. The existence of a vibration induced body contraction behavior shows that the animal perceives mechanical stimuli. Since this behavior is independent of the head, it is likely perceived by specialized mechanosensory receptor neurons associated with the nerve cords.

### Capacity of head and brain regeneration

Certain acoel species display a rather striking regenerative potential similar to planarians. However not all acoels show the same regenerative capacity and the ability to restore lost tissues varies largely between different species. Some acoel species can reproduce asexually by budding and subsequently regenerating the remaining body parts (Sikes and Bely, 2010). For instance, *Isodiametra pulchra* is well able to regenerate the posterior part of the body within 10 days, though it is not capable of regenerating its head (Perea-Atienza et al., 2013). By contrast, *Hofstenia miamia* is capable to regenerate its head as well as its tail (Srivastava et al., 2014). RNAi experiments in *Hofstenia* show that bmp and wnt signaling are required for whole-body regeneration similarly to what has previously been found in planarians. The ability of *Hofstenia* to regenerate its head suggests that also the brain can be restored, however, brain anatomy and the behavior of *Hofstenia* remains largely unexplored. In principle, *S. roscoffensis* shows the capacity to regenerate the head as well as the tail after amputation at different body levels. Ablation studies of the eyes in *Praesagittifera naikaiensis* showed that these structures can regenerate and suggest that photoreceptive capacity of the new eyes are restored as shown by behavioral assays (Yamasu, 1991). While we did not specifically ablate the eyes in *S. roscoffensis* our results show that both eyes and brain can regenerate and that visual behavior is restored. However the recovery of light attraction does not reach the same degree as before decapitation. It will be interesting to investigate if the trend of increasing phototaxis recovery continues after 50 dpa and if the original performance might be reached. Another intriguing question arising from our findings is that the animals do not recover geotaxis, while the statocyst regenerates. It is possible that the neural circuits required for geotaxis do not recover after decapitation or, alternatively, that a longer time period for functional recovery is required. Further analysis of brain regenerations using additional markers, such as neurotransmitters might provide answers to this question. We found that the dSap47 antibody from *Drosophila* cross-reacts in *S. roscoffensis* and *Isodiametra pulchra* (S.G.S and M.B., unpublished), therefore may also cross-react with the Sap47 homolog in other animal species. From RNAseq experiments we have identified the *S. roscoffensis Sap47* transcript, highlighting several conserved domains which in turn may be recognized by the anti-dSap47 antibody (Fig. S2). It will be interesting to test this antibody in animals of other phyla. Moreover, the cellular processes that act during brain regeneration remain unknown. It will be of great interest to assess the temporal processes of brain regeneration and the molecular mechanisms involved. In particular the application of new emerging genetic techniques such as the Cas9/CRISPR system in combination with RNAi or genome sequencing data may provide the necessary tools to study the cellular, genetic and molecular mechanisms acting in the regeneration of a functional brain.

## MATERIALS AND METHODS

### Animal culture and handling

Adult *S. roscoffensis* specimen were collected near the Station Biologique de Roscoff in Brittany (France) and transported in falcon tubes to the Department of Biology at the University of Fribourg. Animals were kept at 19°C under 12/12 h light/dark cycles in glass or plastic (Greiner Bio-One) Petri dishes filled with 34‰ f/2 Medium (Guillard, 1975, modified according to Andersen et al., 2005). Filtered seawater was changed weekly or according to needs. For the decapitation procedure a group of about 10-15 animals were transferred into a 1 ml drop of ASW (artificial sea water) on a silicon plate. Amputation was performed manually using sharpened minutien pins (0.1 mm diameter, Fisher Scientific Tools). Two needles were used to induce a cut between statocyst and mouth opening, in such a fashion removing the entire head and all sensory head organs. One needle was first placed at the appropriate position and thereby used to stabilize the animal, while the second needle was used to cut the body. All animal experiments conformed to the relevant regulatory standards.

### Immunocytochemistry

For the immunohistochemical analysis intact or regenerated adult animals were fixed in 4% Formaldehyde in 0.1 M phosphate buffered solution (PBS) for 30 min in glass wells. After fixation specimen were washed 3×5 min with 1% PBT (Triton X-100 in PBS). Primary antibody incubation was performed overnight at 4°C, glass wells were covered with Parafilm. After primary antibody incubation specimen were washed 3×5 min and 4×30 min with 1% PBT. Secondary antibody incubation was done at room temperature for 2 h or overnight at 4°C. After secondary antibody incubation specimen were washed 3×5 min and 4×30 min with 1% PBT. Specimens were mounted in Vectashield H-1200 (Vector Laboratories).

Primary antibody was mouse anti-dSap47 1:20 (Developmental Studies Hybridoma Bank) (Reichmuth et al., 1995). Secondary antibodies used for confocal microscopic analysis were Alexa Fluor 488, Alexa Fluor 555, and Alexa Fluor 647 antibodies generated in goat (Molecular Probes), all in a 1:300 dilution. Cytological staining for muscles and actin rich structures was done using Phalloidin coupled to Alexa Fluor 647 at 1:10,000 dilution (Life Technologies, A22287). Nuclear stain was done using DAPI (4′, 6-diamino-phenyin-dole), which was contained in the Vectashield mounting medium.

### Laser confocal microscopy and image processing

For laser confocal microscopy, a Leica TCS SPE or Leica TCS SP5 was used. Optical sections ranged from 0.2-1.5 μm recorded in stack average mode with picture size of 1024×1024 pixels. Captured images from optical sections were imported and processed using ImageJ. Generation of 3D digital models, raw tiff stacks (stacks of optical sections) were done using AMIRA (Mercury Computer Systems) as previously described (Sprecher et al., 2007a,b).

### Behavioral phototaxis, geotaxis and vibration induced body contraction assays

For phototaxis experiments a group of about 20 animals was placed in the middle of a plastic Petri dish (60-mm diameter; Greiner Bio-One GmbH, Cat.No. 628103), which was placed on a black printed paper depicting 12 zones (each 0.25 cm ranging from +2.75 cm to −2.75 cm). Lighting regimes were adapted from previously described phototaxis assays: A LED lamp (OSARAM LED, 80012 White) was placed at a 45° angle 9 cm above the table at a distance that the lamp-oriented side of the Petri dish was illuminated at 1100 lux, the middle of the plate 840 lux and the side opposite of the lamp with 400 lux (Keene et al., 2011; von Essen et al., 2011). After 5 min the number of animals in each zone was counted. The temperature was controlled in the experimental chamber throughout all experiments between 23 and 25°C.

For geotaxis experiments individual animals were sucked into a yellow 100 ml pipette tip (Treff lab, Cat.No. 96.01701.4.02). Before the experiment a 1.7 cm distance for the tip of the pipette tip was marked with a water-proof black marker. Animals were let settle behind the marked line. The pipette tip was held at about 45° angle manually and the time from crossing the black line to the tip of the pipette tip was measured for each animal.

For vibration induced body contraction experiments animals were placed in a Petri dish under a stereomicroscope. Once the animal was in the field of view a pipette tip was tapped lightly against the side of the Petri dish. This light tapping elicits a highly stereotypic body contraction response in all animals assessed.

For the series of experiments performed with decapitated animals, the animals were first tested in the phototaxis assay, before testing them for geotaxis and vibration response. Between the assays we let the animals recover in a fresh Petri dish for about 2 h. After all experiments were performed animals were transferred into fresh ASW and placed back in the incubator. To assess statistical significance between groups in the behavioral assays we applied the wilcox signed rank test. All statistical analyses and visualizations were done with R version 3.0.2 (www.r-project.org). Significance levels with a Bonferroni correction for multiple comparisons were: phototaxis and geotaxis in Fig. 5 *P<0.01, **P<0.002 and ***P<0.0002. In Fig. S1 two groups were used therefore the significance levels without a Bonferroni correction were *P<0.05,**P<0.01 and ***P<0.001.

To assess the locomotor ability of the worms, we measured the speed of non-decapitated worms and of worms one day after decapitation. For each experimental group we used 80 worms divided in two Petri dishes (40 worms in each) and allowed them to move freely in the dish for 5 min before recording a 45 s movie under red LEDs to circumvent phototaxis (Nissen et al., 2015) using a Basler camera. All movies were analyzed and the average speed for each track was measured with the wrMTrck plugin for ImageJ.

## References

[BIO014266C1] Achatz J. G. and Martinez P. (2012). The nervous system of Isodiametra pulchra (Acoela) with a discussion on the neuroanatomy of the Xenacoelomorpha and its evolutionary implications. *Front. Zool.* 9, 27 10.1186/1742-9994-9-27 23072457 PMC3488495

[BIO014266C2] Andersen R. A., Berges J. A., Harrison P. J. and Watanabe M. M. (2005). In *Appendix A Recipes for Freshwater and Seawater Media. Algal Culturing Techniques* (ed. Andersen R. A.), pp. 429-538. Amsterdam: Elsevier.

[BIO014266C3] Bailly X., Laguerre L., Correc G., Dupont S., Kurth T., Pfannkuchen A., Entzeroth R., Probert I., Vinogradov S., Lechauve C. et al. (2014). The chimerical and multifaceted marine acoel Symsagittifera roscoffensis: from photosymbiosis to brain regeneration. *Front. Microbiol.* 5, 498 10.3389/fmicb.2014.00498 25324833 PMC4183113

[BIO014266C4] Bery A., Cardona A., Martinez P. and Hartenstein V. (2010). Structure of the central nervous system of a juvenile acoel, Symsagittifera roscoffensis. *Dev. Genes Evol.* 220, 61-76. 10.1007/s00427-010-0328-2 20549514 PMC2929339

[BIO014266C5] Bressan J. M., Benz M., Oettler J., Heinze J., Hartenstein V. and Sprecher S. G. (2014). A map of brain neuropils and fiber systems in the ant Cardiocondyla obscurior. *Front. Neuroanat.* 8, 166 10.3389/fnana.2014.00166 25698935 PMC4316776

[BIO014266C6] Dreyer D., Vitt H., Dippel S., Goetz B., El Jundi B., Kollmann M., Huetteroth W. and Schachtner J. (2010). 3D Standard brain of the red flour beetle tribolium castaneum: a tool to study metamorphic development and adult plasticity. *Front. Syst. Neurosci.* 4, 3 10.3389/neuro.06.003.2010 20339482 PMC2845059

[BIO014266C7] El Jundi B., Heinze S., Lenschow C., Kurylas A., Rohlfing T. and Homberg U. (2009). The locust standard brain: a 3D standard of the central complex as a platform for neural network analysis. *Front. Syst. Neurosci.* 3, 21 10.3389/neuro.06.021.2009 20161763 PMC2818101

[BIO014266C8] Fernandez-Hernandez I. and Rhiner C. (2015). New neurons for injured brains? The emergence of new genetic model organisms to study brain regeneration. *Neurosci. Biobehav. Rev.* 56, 62-72. 10.1016/j.neubiorev.2015.06.021 26118647

[BIO014266C9] Grandel H. and Brand M. (2013). Comparative aspects of adult neural stem cell activity in vertebrates. *Dev. Genes Evol.* 223, 131-147. 10.1007/s00427-012-0425-5 23179636

[BIO014266C10] Guillard R. R. L. (1975). Culture of phytoplankton for feeding marine invertebrates. In *Culture of Marine Invertebrate Animals* (ed. Smith W. L. and Chanley M. H.), pp. 26-60. New York: Plenum Press.

[BIO014266C11] Heinze S. and Reppert S. M. (2012). Anatomical basis of sun compass navigation I: the general layout of the monarch butterfly brain. *J. Comp. Neurol.* 520, 1599-1628. 10.1002/cne.23054 22473804

[BIO014266C12] Keeble F. (1910). *Plant-Animals: A Study in Symbiosis*. Cambridge, UK: Cambridge University Press.

[BIO014266C13] Keene A. C., Mazzoni E. O., Zhen J., Younger M. A., Yamaguchi S., Blau J., Desplan C. and Sprecher S. G. (2011). Distinct visual pathways mediate Drosophila larval light avoidance and circadian clock entrainment. *J. Neurosci.* 31, 6527-6534. 10.1523/JNEUROSCI.6165-10.2011 21525293 PMC3103866

[BIO014266C14] Lepousez G., Nissant A. and Lledo P.-M. (2015). Adult neurogenesis and the future of the rejuvenating brain circuits. *Neuron* 86, 387-401. 10.1016/j.neuron.2015.01.002 25905812

[BIO014266C15] Li Q., Yang H. and Zhong T. P. (2015). Regeneration across metazoan phylogeny: lessons from model organisms. *J. Genet. Genomics* 42, 57-70. 10.1016/j.jgg.2014.12.002 25697100

[BIO014266C16] Nissen M., Shcherbakov D., Heyer A., Brummer F. and Schill R. O. (2015). Behaviour of the plathelminth Symsagittifera roscoffensis under different light conditions and the consequences for the symbiotic algae Tetraselmis convolutae. *J. Exp. Biol.* 218, 1693-1698. 10.1242/jeb.110429 25852067

[BIO014266C17] Perea-Atienza E., Botta M., Salvenmoser W., Gschwentner R., Egger B., Kristof A., Martinez P. and Achatz J. G. (2013). Posterior regeneration in Isodiametra pulchra (Acoela, Acoelomorpha). *Front. Zool.* 10, 64 10.1186/1742-9994-10-64 24160844 PMC3816570

[BIO014266C18] Perea-Atienza E., Gavilan B., Chiodin M., Abril J. F., Hoff K. J., Poustka A. J. and Martinez P. (2015). The nervous system of Xenacoelomorpha: a genomic perspective. *J. Exp. Biol.* 218, 618-628. 10.1242/jeb.110379 25696825

[BIO014266C19] Raikova O. I., Reuter M., Kotikova E. A. and Gustafsson M. K. S. (1998). A commissural brain! The pattern of 5-HT immunoreactivity in Acoela (Plathelminthes). *Zoomorphology* 118, 69-77. 10.1007/s004350050058 9569678

[BIO014266C20] Reichmuth C., Becker S., Benz M., Debel K., Reisch D., Heimbeck G., Hofbauer A., Klagges B., Pflugfelder G. O. and Buchner E. (1995). The sap47 gene of Drosophila melanogaster codes for a novel conserved neuronal protein associated with synaptic terminals. *Brain Res. Mol. Brain Res.* 32, 45-54. 10.1016/0169-328X(95)00058-Z 7494462

[BIO014266C21] Reuter M., Raikova O. I. and Gustafsson M. K. S. (1998). An endocrine brain? The pattern of FMRF-amide immunoreactivity in Acoela (Plathelminthes). *Tissue Cell* 30, 57-63. 10.1016/S0040-8166(98)80006-2 9569678

[BIO014266C22] Reuter M., Raikova O. I., Jondelius U., Gustafsson M. K. S., Maule A. G. and Halton D. W. (2001). Organisation of the nervous system in the Acoela: an immunocytochemical study. *Tissue Cell* 33, 119-128. 10.1054/tice.2000.0134 11392663

[BIO014266C23] Semmler H., Chiodin M., Bailly X., Martinez P. and Wanninger A. (2010). Steps towards a centralized nervous system in basal bilaterians: insights from neurogenesis of the acoel Symsagittifera roscoffensis. *Dev. Growth Differ.* 52, 701-713. 10.1111/j.1440-169X.2010.01207.x 20874714

[BIO014266C24] Sikes J. M. and Bely A. E. (2010). Making heads from tails: development of a reversed anterior-posterior axis during budding in an acoel. *Dev. Biol.* 338, 86-97. 10.1016/j.ydbio.2009.10.033 19878663

[BIO014266C25] Silver J., Schwab M. E. and Popovich P. G. (2015). Central nervous system regenerative failure: role of Oligodendrocytes, Astrocytes, and Microglia. *Csh Perspect. Biol.* 7, a020602 10.1101/cshperspect.a020602 PMC4355267 25475091

[BIO014266C26] Sprecher S. G., Pichaud F. and Desplan C. (2007a). Adult and larval photoreceptors use different mechanisms to specify the same Rhodopsin fates. *Genes Dev.* 21, 2182-2195. 10.1101/gad.1565407 17785526 PMC1950857

[BIO014266C27] Sprecher S. G., Reichert H. and Hartenstein V. (2007b). Gene expression patterns in primary neuronal clusters of the Drosophila embryonic brain. *Gene Expr. Patterns* 7, 584-595. 10.1016/j.modgep.2007.01.004 17300994 PMC3928073

[BIO014266C28] Srivastava M., Mazza-Curll K. L., van Wolfswinkel J. C. and Reddien P. W. (2014). Whole-body Acoel regeneration is controlled by Wnt and Bmp-Admp signaling. *Curr. Biol.* 24, 1107-1113. 10.1016/j.cub.2014.03.042 24768051

[BIO014266C29] Tanaka E. M. and Reddien P. W. (2011). The cellular basis for animal regeneration. *Dev. Cell* 21, 172-185. 10.1016/j.devcel.2011.06.016 21763617 PMC3139400

[BIO014266C30] Todt C. and Tyler S. (2007). Ciliary receptors associated with the mouth and pharynx of Acoela (Acoelomorpha): a comparative ultrastructural study. *Acta Zool.* 88, 41-58. 10.1111/j.1463-6395.2007.00246.x

[BIO014266C31] von Essen A. M. H. J., Pauls D., Thum A. S. and Sprecher S. G. (2011). Capacity of visual classical conditioning in Drosophila larvae. *Behav. Neurosci.* 125, 921-929. 10.1037/a0025758 21967373

[BIO014266C32] Yamasu T. (1991). Fine structure and function of Ocelli and Sagittocysts of Acoel flatworms. *Hydrobiologia* 227, 273-282. 10.1007/BF00027612

